# Modeling of One-Dimensional Thermoelastic Dual-Phase-Lag Skin Tissue Subjected to Different Types of Thermal Loading

**DOI:** 10.1038/s41598-020-60342-6

**Published:** 2020-02-25

**Authors:** Hamdy M. Youssef, Najat A. Alghamdi

**Affiliations:** 1 Engineering Mechanics Department-College of Engineering and Islamic Architecture -Umm Al-Qura University-, Makkah, Saudi Arabia; 2 Mathematics Department-Faculty of Applied Science-Umm Al-Qura University-, Makkah, Saudi Arabia

**Keywords:** Computational biophysics, Biomedical engineering

## Abstract

This work introduces a mathematical model of thermoelastic skin tissue in the context of the dual-phase-lag heat conduction law. One-dimensional skin tissue has been considered with a small thickness and its outer surface traction free. The bounding plane of the skin tissue is subjected to three different types of thermal loading; thermal shock, ramp type heating, and harmonic heating. The inner surface has no temperature increment and traction free. Laplace transform techniques have been used, and its inversions have been calculated by using the Tzuo method. The numerical results have been represented in figures. The thermal shock time parameter, the ramp-type heat parameter, and the angular thermal parameter have significant effects on the temperature increment, the strain, the displacement, and the stress distributions, and they play vital roles in the speed propagation of the thermomechanical waves through the skin tissue.

## Introduction

The essential challenge in thermal therapy is distributing adequate heat to a target tissue without influential surrounding tissues. Medically, various thermal therapies are widespread used to treat disease and injury involving skin tissue, where thermally induced within the infected tissue but without affecting the healthy tissue. Thus, an urgent need is to figure out how the temperature and stress fields impact the kinetics of thermal treatment. Accordingly, accurate predictions of thermal and mechanical responses in biological tissue are essential for designing new clinical thermal systems. Some researches explained that tissue deformation due to heating and cooling might produce pain sensation^1,2^.

Thermal analysis of heat transfer through thermoelastic skin tissue is essential for many therapy applications^3^. However, it would be much better if we could understand the biomechanics associated with them before a medical action is applied. One of the hardships in biomechanics is to specify the mechanical properties of materials and tissues understudying^4^. Pennes^5^ was first recognized the bioheat transfer equation to model the temperature in the human forearm, and other researches established bioheat transfer theorems^6–8^.

It is observed that even a small increment of heat-induced stress can destroy the immune response; protein cell organelle structures can be changed, resulting in cell death^1^. Most studies emphasis on heat conduction^9–20^, while the heating which induced deformation is not considered. Tunc^21^ solved the bioheat transfer equation considering variable blood perfusion values and the temperature field in the context of the Pennes’s model. Xu *et al*.^22,23^ discussed the heat transfer, thermal damage, and stress due to the heat of the human skin. Shen *et al*.^24^ used a thermomechanical model to study the thermomechanical interaction of skin tissue at a high temperature. Kim *et al*.^25^ discussed the thermal and mechanical effects due to pulsed laser absorption in the human skin.

The generalized thermoelastic theories have been applied in solving transient thermal shock problems. Glass *et al*. presented an analytic solution for a linear heat conduction problem in a semi-infinite medium influenced by a periodic on-off type heat flux^26^. Moreover, he studied the non-linear case by adding the effect of surface radiation into an external ambient. Lord and Shulman^27^ formulated a generalized dynamical theory of thermoelasticity with one relaxation time using a form of the heat transport equation. Green and Lindsay have developed a different thermoelastic theory by introducing two relaxation time into the constitutive equations^28^. McBride, Andrew, *et al*. constructed thermoelastic modeling of the skin at finite deformations^29^. Li *et al*. introduced an analytical study of transient thermomechanical responses of dual-layer skin tissue with variable thermal conductivity^30^.

## Formulation of the Problem

Tzou suggested the DPL model solve the problems that occurred in the classical heat flux model as^31^:

1 $$q(x,t+{\tau }_{q})=-\,K\nabla T(x,t+{\tau }_{T})$$

where *T* is the absolute temperature, *K* is the thermal conductivity constant, *t* is the time variable, and ${\tau }_{q},{\tau }_{T}$ are the phase-lag parameters of the heat flux and the phase-lag of the temperature gradient, respectively. Generally, the relaxation times $\{{\tau }_{q},{\tau }_{T}\}\ge 0$ take minimal values, while in the biological materials, those parameters are significant.

The equation of energy conservation of bioheat transfer can be described as^31^:

2 $$\rho C\frac{\partial T}{\partial t}=-\,\nabla \cdot q-{W}_{b}{C}_{b}{\rho }_{p}(T-{T}_{b})+({Q}_{met}+{Q}_{ext})$$

where $\rho$ is the density, $C$is the specific heat, ${C}_{b}$ and ${W}_{b}$ are the specific heat and perfusion rate of blood, respectively. ${Q}_{met}$ is the metabolic heat generation, ${Q}_{ext}$ is the external heat source and ${T}_{b}$ is the arterial temperature.

The DPL model based on two effects; the heat flux $q\,$and the gradient of the temperature $\nabla T$, which modified the classical Fourier’s law of heat conduction. It gives the following heat conduction equation.

3 $$\begin{array}{c}(1+{\tau }_{T}\frac{\partial }{\partial t})\frac{{\partial }^{2}T}{\partial {x}^{2}}=\frac{\rho C}{K}(1+{\tau }_{q}\frac{\partial }{\partial t})\frac{\partial T}{\partial t}+\frac{{w}_{b}{C}_{b}{\rho }_{p}}{K}(1+{\tau }_{q}\frac{\partial }{\partial t})(T-{T}_{b})\\ +\frac{\gamma {T}_{0}}{K}(1+{\tau }_{q}\frac{\partial }{\partial t})e-\frac{1}{K}(1+{\tau }_{q}\frac{\partial }{\partial t})({Q}_{met}+{Q}_{ext})\end{array}$$

Because of the chemical reactions, the metabolic heat source within the tissues is valid, and it is assumed to take constant value ${Q}_{met}=368.1W/{m}^{3}$. With zero value, the external heat source will be assumed; thus, ${Q}_{ext}=0$^5,32,33^.

Hence, the heat conduction equation takes the form:

4 $$(1+{\tau }_{T}\frac{\partial }{\partial t})\frac{{\partial }^{2}\theta }{\partial {x}^{2}}=\frac{\rho C}{K}(1+{\tau }_{q}\frac{\partial }{\partial t})\frac{\partial \theta }{\partial t}+\frac{{w}_{b}{C}_{b}{\rho }_{p}}{K}(1+{\tau }_{q}\frac{\partial }{\partial t})\theta +\frac{\gamma {T}_{0}}{K}(1+{\tau }_{q}\frac{\partial }{\partial t})e-\frac{{Q}_{met}}{K}$$

where $\theta =(T-{T}_{b})\,$is the temperature increment.

The equation of motion of a one-dimensional thermoelastic material is^30^:

5 $$(\lambda +2\mu )\frac{{\partial }^{2}e}{\partial {x}^{2}}-\gamma \frac{{\partial }^{2}\theta }{\partial {x}^{2}}=\rho \frac{{\partial }^{2}e}{\partial {t}^{2}}$$

The stress-strain relation in the form:

6 $$\sigma =(\lambda +2\mu )e-\gamma \theta $$

The displacement $u=u(x,t)$ satisfies the relation:

7 $$e=\frac{\partial u}{\partial x}$$

**Figure 1:**
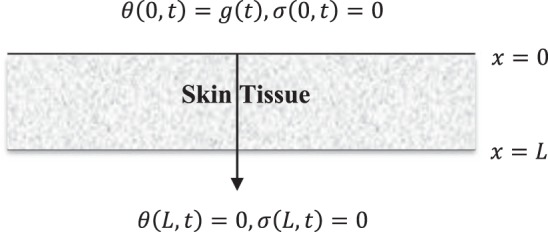
The skin tissue model.

We consider that the outer surface of the skin tissue is subjected to thermal loading and traction free while the inner surface has no temperature increment and traction free also, which gives

8 $$\theta (0,t)=g(t),\theta (L,0)=0$$

and

9 $$\sigma (0,t)=0,\sigma (L,t)=0$$

where $g(t)\,$is the thermal loading function on the outer surface of the skin tissue $x=0$ as in Fig. 1:

To simplify the governing equations, we will use the following non-dimensional variables Youssef [34]:

$$\{x{\prime} ,u{\prime} ,L{\prime} \}={c}_{0}\eta \{x,u,L\},\{t{\prime} ,{t}_{0}^{{\prime} },{\tau }_{T}^{{\prime} },{\tau }_{q}^{{\prime} }\}={c}_{0}^{2}\eta \{t,{t}_{0},{\tau }_{T},{\tau }_{q}\},\theta {\prime} =\frac{\theta }{{T}_{0}}\sigma {\prime} =\frac{\sigma }{\lambda +2\mu },Q{\prime} =\frac{Q}{{T}_{0}}.$$

where ${c}_{0}=\sqrt{\frac{\lambda +2\mu }{\rho }}$ is the longitudinal wave speed parameter and $\eta =\frac{\rho C}{K}$ is the thermal viscosity parameter.

Hence, we obtain

10 $$(1+{\tau }_{T}\frac{\partial }{\partial t})\frac{{\partial }^{2}\theta }{\partial {x}^{2}}=(1+{\tau }_{q}\frac{\partial }{\partial t})\frac{\partial \theta }{\partial t}+{\beta }_{1}(1+{\tau }_{q}\frac{\partial }{\partial t})\theta +{\beta }_{2}(1+{\tau }_{q}\frac{\partial }{\partial t})e-Q$$

11 $$\frac{{\partial }^{2}e}{\partial {x}^{2}}-{\beta }_{3}\frac{{\partial }^{2}\theta }{\partial {x}^{2}}=\frac{{\partial }^{2}e}{\partial {t}^{2}}$$

and

12 $${\sigma }_{xx}=e-{\beta }_{3}\theta $$

where

$${\beta }_{1}=\frac{{w}_{b}{C}_{b}{\rho }_{p}}{{c}_{o}^{2}{\eta }^{2}K},{\beta }_{2}=\frac{\gamma }{{c}_{o}^{2}{\eta }^{2}K},Q=\frac{{Q}_{met}}{{c}_{o}^{2}{\eta }^{2}K},{\beta }_{3}=\frac{\gamma {T}_{0}}{\lambda +2\mu }.$$

Applying Laplace transform which is defined as:

13 $$\bar{f}(s)=L[f(t)]={\int }_{0}^{\infty }f(t){e}^{-st}dt$$

Hence, Eqs. (10)–(12) and (7) take the forms:

14 $$\frac{{d}^{2}\bar{\theta }}{d{x}^{2}}-{a}_{1}\bar{\theta }={a}_{2}\bar{e}-\tilde{Q}$$

15 $$\frac{{d}^{2}\bar{e}}{d{x}^{2}}-{s}^{2}\bar{e}={\beta }_{3}\frac{{d}^{2}\bar{\theta }}{d{x}^{2}}$$

16 $$\bar{\sigma }=\bar{e}-{\beta }_{3}\bar{\theta }$$

and

17 $$\bar{e}=\frac{d\bar{u}}{dx}$$

While applying the Laplace transform, we assumed all the state-functions have zero-initial value as:

18 $$\theta (x,0)=e(x,0)=\frac{\partial \theta (x,0)}{\partial t}=\frac{\partial e(x,0)}{\partial t}=0$$

After applying the Laplace transform, the boundary conditions (8) and (9) take the forms:

19 $$\bar{\theta }(0,s)=\bar{g}(s),\bar{\theta }(L,s)=0$$

and

20 $$\bar{\sigma }(0,s)=0,\bar{\sigma }(L,s)=0$$

$$where\,{a}_{1}=\frac{(1+{\tau }_{q}s)(s+{\beta }_{1})}{(1+{\tau }_{T}s)},{a}_{2}=\frac{(1+{\tau }_{q}s){\beta }_{2}}{(1+{\tau }_{T}s)},\tilde{Q}=\frac{Q}{s(1+{\tau }_{T}s)}$$

Eliminating $\bar{e}$ between Eqs. (14) and (15), we get

21 $$[{D}^{4}-\ell {D}^{2}+m]\,\bar{\theta }(x,s)={s}^{2}\tilde{Q}$$

Eliminating $\bar{\theta }$ between Eqs. (14) and (15), we obtain

22 $$[{D}^{4}-\ell {D}^{2}+m]\,\bar{e}(x,s)=0$$

where

$$\ell ={a}_{1}+{s}^{2}+{a}_{2}{\beta }_{3},m={a}_{1}{s}^{2}.$$

The general solution of the Eq. (17) takes the form

23 $$\bar{\theta }(x,s)=\mathop{\sum }\limits_{i=1}^{2}({\varepsilon }_{i}{e}^{-{k}_{i}x}+{\eta }_{i}{e}^{{k}_{i}x})+\frac{\tilde{Q}}{{a}_{1}},0\le x\le L$$

The general solution of the Eq. (18) takes the form

24 $$\bar{e}(x,s)=\frac{1}{{a}_{2}}\mathop{\sum }\limits_{i=1}^{2}({k}_{i}^{2}-{a}_{1})({\varepsilon }_{i}{e}^{-{k}_{i}x}+{\eta }_{i}{e}^{{k}_{i}x}),0\le x\le L$$

where ${\varepsilon }_{1},{\varepsilon }_{2},{\eta }_{1},{\eta }_{2}$ are some constants and $\pm {k}_{1},\pm {k}_{2}$ are the roots of the characteristic equation:

25 $${k}^{4}-\ell {k}^{2}+m=0$$

Applying the boundary conditions in (19) on Eq. (23), hence, we get

26 $$\mathop{\sum }\limits_{i=1}^{2}({\varepsilon }_{i}+{\eta }_{i})={\alpha }_{1}$$

27 $$\mathop{\sum }\limits_{i=1}^{2}({\varepsilon }_{i}{e}^{-{k}_{i}L}+{\eta }_{i}{e}^{{k}_{i}L})=-\,\frac{\tilde{Q}}{{a}_{1}}$$

To apply the boundary conditions on (20), we re-write the Eq. (16) as follows:

28 $$\bar{e}=\bar{\sigma }+{\beta }_{3}\bar{\theta }$$

Then, the mechanical boundary conditions (20) have been modified as:

29 $$\bar{e}(0,s)={\alpha }_{2},\bar{e}(L,s)=0$$

where

$${\alpha }_{1}=\bar{g}(s)-\frac{\tilde{Q}}{{a}_{1}},{\alpha }_{2}={\beta }_{3}\,\bar{g}(s)$$

Applying the boundary condition (29) in the Eq. (24), we obtain

30 $$\mathop{\sum }\limits_{i=1}^{2}({k}_{i}^{2}-{a}_{1})({\varepsilon }_{i}+{\eta }_{i})={a}_{2}{\alpha }_{2}$$

and

31 $$\mathop{\sum }\limits_{i=1}^{2}({k}_{i}^{2}-{a}_{1})({\varepsilon }_{i}{e}^{-{k}_{i}L}+{\eta }_{i}{e}^{{k}_{i}L})=0$$

By solving the system of linear Eqs. (26), (27), (30) and (31), we complete the solutions in the Laplace transform domain.

To obtain the complete solutions in the Laplace transform domain, we have to determine the function $g(t)$, so we will consider three types of thermal loading as follows:

1- The thermal shock

32 $$g(t)={\theta }_{0}H(t-\nu ),t\ge \nu $$

2- Ramp-type heating

33 $$g(t)={\theta }_{0}[\begin{array}{cc}\frac{t}{{t}_{0}} & 0 < t < {t}_{0}\\ 1 & t\ge {t}_{0}\end{array}]$$

3- Harmonic thermal heat

34 $$g(t)={\theta }_{0}\,\sin (\omega t),t\ge 0$$

where ${\theta }_{0} > 0$ is constant, which gives the strength of the thermal loading, $H(\ast )$ is the Heaviside unit step function, $\nu \ge 0$ is the thermal shock parameter, ${t}_{0} > 0\,$is the ramping time parameter, and $\omega  > 0$ is the angular thermal loading parameter.

Applying Laplace transform to the Eqs. (32)–(34), we obtain

35 $$\bar{g}(s)=[\begin{array}{cc}\frac{{\theta }_{0}{e}^{-\upsilon s}}{s} & {\rm{for}}\,{\rm{thermal}}\,{\rm{shock}}\\ \frac{{\theta }_{0}(1-{e}^{-{t}_{0}s})}{{t}_{0}{s}^{2}} & {\rm{for}}\,{\rm{ramp}}\,{\rm{type}}\,{\rm{heat}}\\ \frac{{\theta }_{0}\omega }{{s}^{2}-{\omega }^{2}} & {\rm{for}}\,{\rm{harmoinc}}\,{\rm{heat}}\end{array}]$$

## The Numerical Results and Discussions

The Riemann-sum approximation method is used to get the inversion of the Laplace transform. In the Tzou method, any function in the Laplace domain can be inverted to the time domain as^31^.

36 $$g(t)=\frac{{e}^{\kappa t}}{t}[\frac{1}{2}(\kappa )+\mathrm{Re}\mathop{\sum }\limits_{n=1}^{N}{(-1)}^{n}\bar{g}(\kappa +\frac{n\pi I}{t})],$$

where *Re* is the real part and $I=\sqrt{-1}$ is the imaginary number unit.

For rapid convergence, several numerical experiments have approved that the value $\kappa$ satisfies the relation $\kappa t\approx 4.7$^31^.

The values of the relevant thermal parameters which have been used in the present calculations are in Table 1 as following^4,16,19,29,31^:

**Table 1:** The material properties of the skin tissue.

Parameter	Unit	Skin Tissue
$K$	$W/m\,^\circ C$	0.628
$\rho$	$kg/{m}^{3}$	1000
${\rho }_{b}$	$kg/{m}^{3}$	1060
$C$	$J/kg\,^\circ C$	4187
${C}_{b}$	$J/kg\,^\circ C$	3860
${W}_{b}$	$ml/Cm$	0.00187
${T}_{b}$	$^\circ C$	37
t	s	0.05
${\tau }_{q}$	s	0.02
${\tau }_{T}$	s	0.04

**Figure 2:**
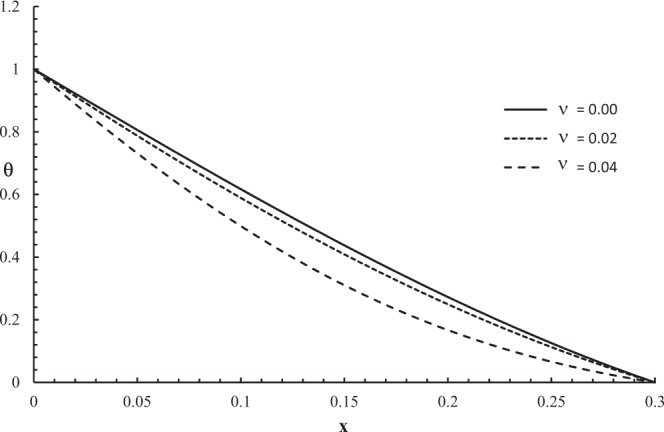
The temperature increment distribution with different values of the thermal shock parameter.

**Figure 3:**
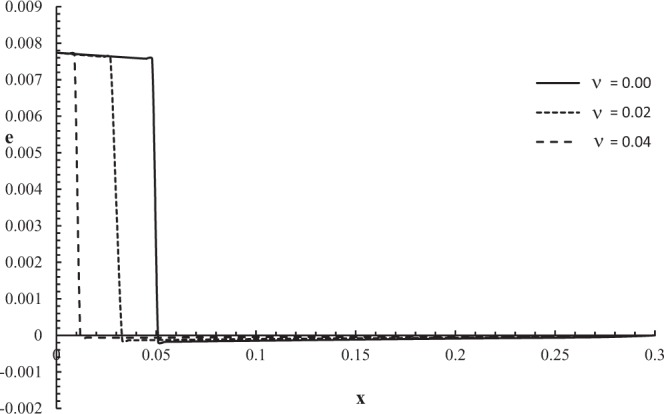
The strain distribution with different values of the thermal shock parameter.

**Figure 4:**
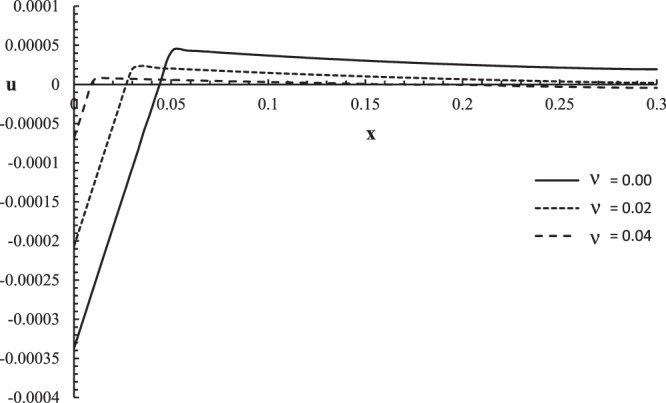
The displacement distribution with different values of the thermal shock parameter.

**Figure 5:**
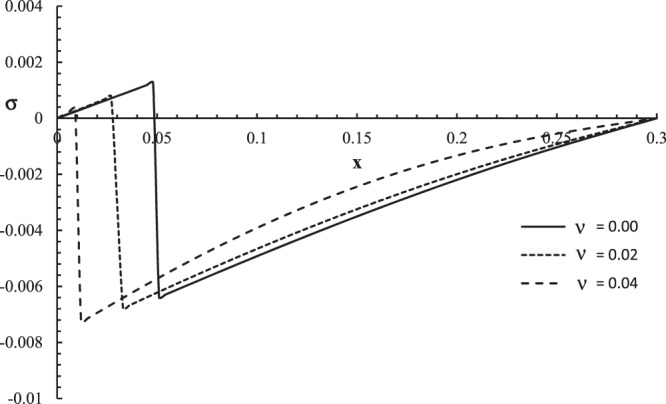
The stress distribution with different values of the thermal shock parameter.

Figures 2–5 represent the temperature increment, the strain, the displacement, and the stress distributions, respectively, with respect to dimensionless length $x=L$ with range $0\le x\le 0.3$ when the dimensionless time $t=0.05$ and the dimensionless relaxation times ${\tau }_{q}=0.02,{\tau }_{T}=0.04$ for various values of dimensionless thermal shock time parameter $\upsilon =(0.0,0.02,0.04)\,$and ${\theta }_{0}=1.0$.

Figure 2 shows that the thermal shock time parameter has a significant effect on the temperature increment distribution. The values of the temperature increment of the three cases are equal to one when $x=0$, and the values of the temperature increment go to zero at the other end of the skin tissue when $x=L$ which agrees with the thermal boundary conditions. The thermal wave has a finite speed of propagation, which agrees with the physical properties of the skin tissue. The value of the temperature increment decreases when the value of the thermal shock time parameter increases.

Figure 3 shows that the thermal shock time parameter has a significant effect on the strain distribution. For the three cases, the values of strain are equal on the bounding plane $x=0$ of the skin tissue $e(0,0.05)=0.00773$, and the values of the strain go to zero $e(L,0.05)=0.0\,$at the other end of the skin tissue $x=L$ which agrees with the mechanical boundary conditions. The mechanical wave has a finite speed of propagation, which agrees with the physical properties of the skin tissue. The absolute value of the strain decreases when the value of the thermal shock time parameter increases. The jump points are ${e(0.045,0.05)|}_{\upsilon =0.0}=0.00757$ ${e(0.027,0.05)|}_{\upsilon =0.02}=0.00761$, and ${e(0.009,0.05)|}_{\upsilon =0.04}=0.00769$.

Figure 4 shows that the thermal shock time parameter has a significant effect on the displacement distribution. The values of the displacement are not equal on the bounding plane of the skin tissue ${u(0,0.05)|}_{\upsilon =0.0}=-\,0.000314$, ${u(0,0.05)|}_{\upsilon =0.02}=-\,0.000183$, and ${u(0,0.05)|}_{\upsilon =0.04}=-\,0.000046$. The displacement distribution has one peak point for each curve, ${u(0.054,0.05)|}_{\upsilon =0.0}=0.000454$, ${u(0.036,0.05)|}_{\upsilon =0.02}=0.000236$, and *u*(0.069, 0.05)${|}_{\upsilon =0.04}=$ 0.0000047. The absolute value of the displacement decreases when the value of the thermal shock time parameter increases.

Figure 5 shows that the thermal shock time parameter has a significant effect on stress distribution. The values of stress are equal to zero on the bounding plane of the skin tissue when$\,x=0$, and the values of the strain go to zero at the other end of the skin tissue when $x=L$ which agrees with the mechanical boundary conditions. The mechanical wave has a finite speed of propagation, which agrees with the physical properties of the skin tissue. The absolute value of the stress decreases when the value of the thermal shock time parameter increases. The jump points of the stress distributions are ${\sigma (0.045,0.05)|}_{\upsilon =0.0}=0.00118$ ${\sigma (0.024,0.05)|}_{\upsilon =0.02}=0.00077$, and ${\sigma (0.006,0.05)|}_{\upsilon =0.04}=0.00023$.

The results which have been shown in Figs. 2–5 agree with the results of the paper^30^.

**Figure 6:**
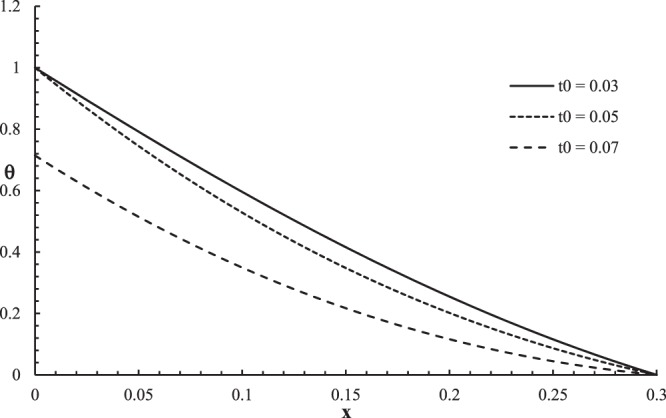
The temperature increment distribution with different values of ramp time parameter.

**Figure 7:**
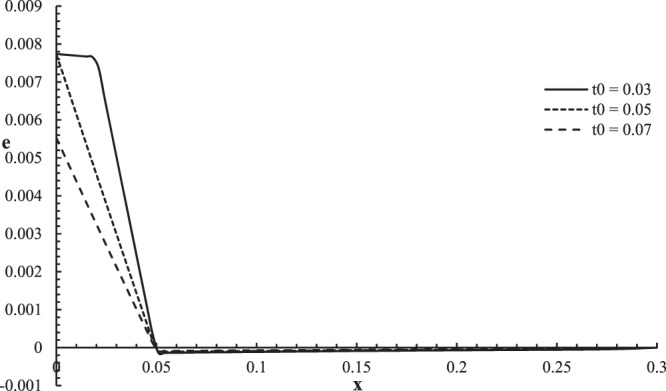
The strain distribution with different values of ramp time parameter.

**Figure 8:**
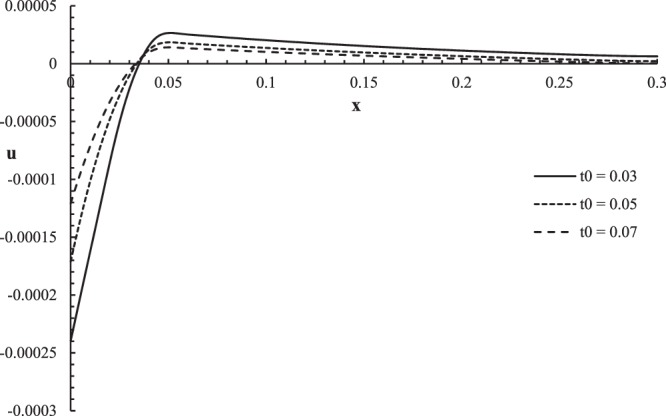
The displacement distribution with different values of ramp time parameter.

**Figure 9:**
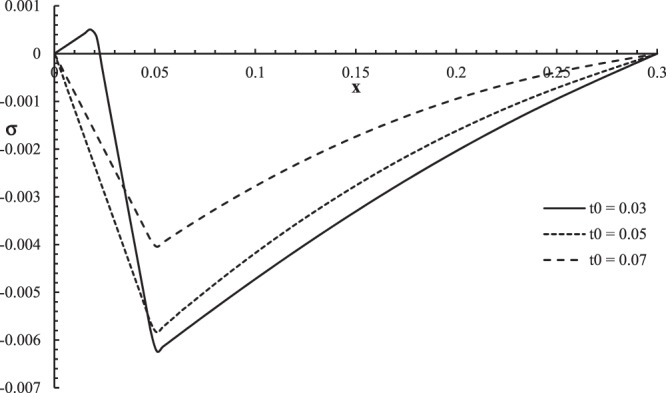
The stress distribution with different values of ramp time parameter.

Figures 6–9 represent the temperature increment, the strain, the displacement, and the stress distributions, respectively, with respect to dimensionless length $x=L$ with range $0\le x\le 0.3$ when the dimensionless time $t=0.05$ and the dimensionless relaxation times ${\tau }_{q}=0.02,{\tau }_{T}=0.04$ for various values of dimensionless ramp-type heat parameter ${t}_{0}=(0.03,0.05,0.07)\,$and ${\theta }_{0}=1.0$ .

Figure 6 shows that the ramp-type heat parameter has a significant effect on the temperature increment distribution. For the two cases $t > {t}_{0}$ and $t={t}_{0}$, the temperature increments are equal to one when $x=0$ of the skin tissue ${\theta (0,0.05)|}_{{t}_{0} < t}={\theta (0,0.05)|}_{{t}_{0}=t}=1.0$, while the temperature increment is less than one ${\theta (0,0.05)|}_{{t}_{0} > t}=0.7$, which agrees with the thermal boundary condition on this side. The values of the temperature increment go to zero $\theta (L,0.05)=0.0\,$at the other end of the skin tissue $x=L$ for all the values of the ramp-type heat parameter, which agrees with the thermal boundary condition on this side. This figure assures that the thermal wave has a finite speed of propagation. The value of the temperature increment decreases when the value of the ramp-type heat parameter increases.

Figure 7 shows that the ramp-type heat parameter has a significant effect on the strain distribution. For the two curves of the cases $t > {t}_{0}$ and $t={t}_{0}$, the values of strain are equal on the bounding plane of the skin tissue ${e(0,0.05)|}_{{t}_{0} < t}={e(0,0.05)|}_{{t}_{0}=t}=0.00726$, while for the case $t < {t}_{0}$ the value of the strain is different ${e(0,0.05)|}_{{t}_{0} > t}=0.00518$, which agrees with the mechanical boundary condition when $x=0$. The values of the strain go to zero $e(L,0.05)=0.0\,$at the other end of the skin tissue $x=L$ which agrees with the mechanical boundary condition on this side. The mechanical wave has a finite speed of propagation, which agrees with the physical properties of the skin tissue. The absolute value of the strain decreases when the value of the ramp-type heat parameter increases. The jump point occurs only for the curve of the case ${t}_{0} < t$ ${e(0.015,0.05)|}_{{t}_{0}=0.3}=0.00765$.

Figure 8 shows that the ramp-type heat parameter has a significant effect on the displacement distribution. The values of displacement are not equal on the bounding plane $x=0$ of the skin tissue ${u(0,0.05)|}_{{t}_{0}=0.03}=-\,0.00022$, ${u(0,0.05)|}_{{t}_{0}=0.05}=-\,0.00015$, and ${u(0,0.05)|}_{{t}_{0}=0.07}=-\,0.00010$. Each curve of the displacement distribution has one peak point which are ${u(0.048,0.05)|}_{{t}_{0}=0.03}=0.000026$, ${u(0.045,0.05)|}_{{t}_{0}=0.05}=0.000017$, and *u*(0.042, 0.05) ${|}_{{t}_{0}=0.07}=0.000012$. The absolute value of the displacement decreases when the value of the ramp-type heat parameter increases.

Figure 9 shows that the ramp-type heat parameter has a significant effect on stress distribution. The values of the stress distribution are equal to zero on the bounding plane $x=0$ of the skin tissue, and the values of the stress go to zero at the other side of the skin tissue, $x=L$ which agrees with the mechanical boundary conditions on both sides. The mechanical wave has a finite speed of propagation, which agrees with the physical properties of the skin tissue. The absolute value of the stress decreases when the value of the ramp-type heat parameter increases. The jump point of the stress distributions is, ${\sigma (0.018,0.05)|}_{{t}_{0}=0.03}=0.0005$ and the peak points are ${\sigma (0.050,0.05)|}_{{t}_{0}=0.05}=-\,0.006$, and ${\sigma (0.051,0.05)|}_{{t}_{0}=0.07}=-\,0.004$.

**Figure 10:**
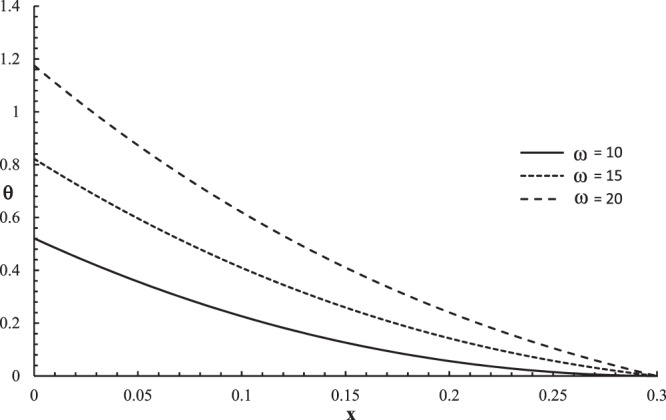
The temperature increment distribution with different values of the angular thermal parameter.

**Figure 11:**
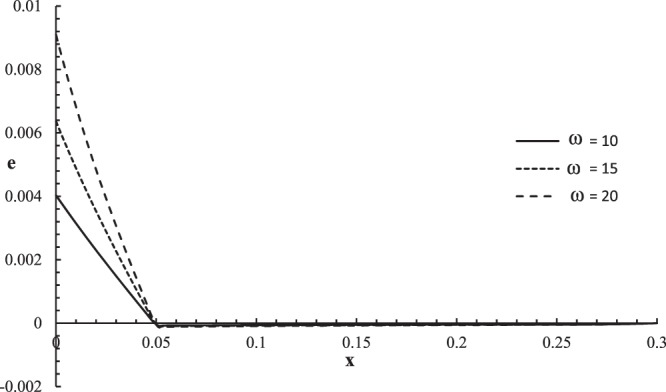
The strain distribution with different values of the angular thermal parameter.

**Figure 12:**
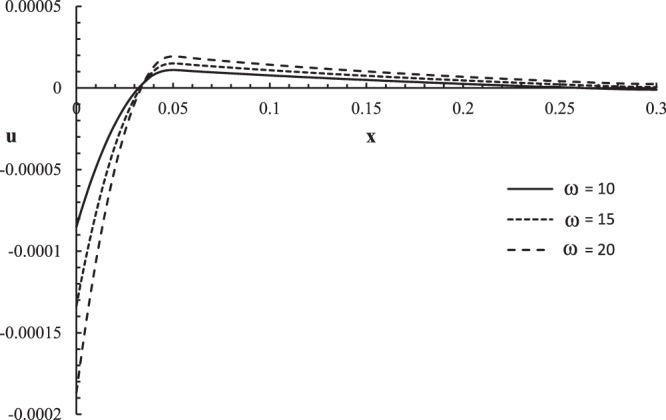
The displacement distribution with different values of the angular thermal parameter.

**Figure 13:**
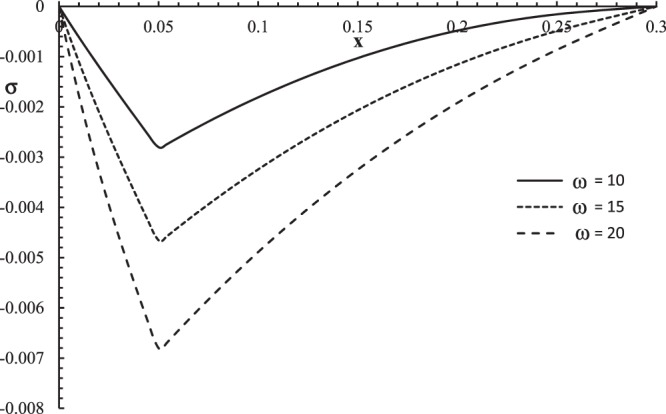
The stress distribution with different values of the angular thermal parameter.

Figures 10–13 represent the temperature increment, the strain, the displacement, and the stress distributions, respectively, with respect to dimensionless length $x=L$ with range $0\le x\le 0.3$ when the dimensionless time $t=0.05$ and the dimensionless relaxation times ${\tau }_{q}=0.02,{\tau }_{T}=0.04$ for various values of the dimensionless angular thermal parameter $\omega =(10,15,20)\,$and ${\theta }_{0}=1.0$ .

Figure 10 shows that the angular thermal parameter has a significant effect on the temperature increment distribution. The three curves have different values on the bounding plane $x=0$ of the skin tissue ${\theta (0,0.05)|}_{\omega =10}=0.51$, ${\theta (0,0.05)|}_{\omega =15}=0.81$, and ${\theta (0,0.05)|}_{\omega =10}=1.16$, which agrees with the thermal boundary condition when on this side. The values of the temperature increment go to zero $\theta (L,0.05)=0.0\,$at the other end of the skin tissue $x=L$ for all the values of the angular thermal parameter, which agrees with the thermal boundary condition on this side. This figure assures that the thermal wave has a finite speed of propagation. The value of the temperature increment increases when the value of the angular thermal parameter increases.

Figure 11 shows that the angular thermal parameter has a significant effect on the strain distribution. The values of strain are not equal on the bounding plane $x=0$ of the skin tissue ${e(0,0.05)|}_{\omega =10}=0.0037$, ${e(0,0.05)|}_{\omega =15}=0.0062$, and ${e(0,0.05)|}_{\omega =20}=0.0092$, which agrees with the mechanical boundary condition. The values of the strain go to zero $e(L,0.05)=0.0\,$at the other end of the skin tissue $x=L$, which agrees with the mechanical boundary condition on this side. The mechanical wave has a finite speed of propagation, which agrees with the physical properties of the skin tissue. The absolute value of the strain increases when the value of the angular thermal parameter increases. All the peak points occur for the three curves in the same position with the same value $e(0.051,0.05)=-\,0.00011$.

Figure 12 shows that the angular thermal parameter has a significant effect on the displacement distribution. The values of displacement are not equal on the bounding plane $x=0$ of the skin tissue ${u(0,0.05)|}_{\omega =10}=-\,0.00007$, ${u(0,0.05)|}_{\omega =15}=-\,0.00012$, and ${u(0,0.05)|}_{\omega =20}=-0.00016$. The displacement distribution has one peak point for each curve, ${u(0.045,0.05)|}_{\omega =10}=0.000015$, ${u(0.048,0.05)|}_{\omega =15}=0.000018$, and *u*(0.042, 0.05) ${|}_{\omega =20}=$ 0.000019. The absolute value of the displacement increases when the value of the angular thermal parameter increases.

Figure 13 shows that the angular thermal parameter has a significant effect on stress distribution. The values of stress are equal to zero on the bounding plane $x=0$ of the skin tissue, and the values of the stress go to zero at the other end of the skin tissue $x=L$, which agrees with the mechanical boundary conditions. The mechanical wave has a finite speed of propagation. The absolute value of the stress increases when the value of the angular thermal parameter increases. The peak points of the stress distributions are ${\sigma (0.048,0.05)|}_{\omega =10}=-\,0.0027$ ${\sigma (0.050,0.05)|}_{\omega =15}=-\,0.0047$, and ${\sigma (0.051,0.05)|}_{\omega =20}=-\,0.0068$.

## Conclusion

A mathematical model of skin tissue has been constructing in the context of dual-phase-lag thermoelasticity. The bounding surface of the tissue traction free and is subjected to three different types of thermal loading (thermal shock, ramp-type heating, and harmonic heating).

The thermal shock time parameter, the ramp-type heat parameter, and the angular thermal parameter have significant effects on the temperature increment, the strain, the displacement, and the stress distributions. The three parameters of the three different types of thermal loading can be used as a controller on the propagation of the thermo-mechanical waves through the thermoelastic skin tissues.

The values of the studied functions decrease when the values of the thermal shock time parameter and the ramp-type heat parameter increase, and when the value of the angular thermal parameter decreases.

The results of this work, especially the thermal shock loading, agree with the results of the work^4,30^ and agree with the results of many other work rather than skin tissue^34,35^.
